# Protocol for a Systematic Review and Meta-Analysis of Observational Studies on the Association of Exposure to Toxic Environmental Pollutants and Left Ventricular Dysfunction

**DOI:** 10.3390/ijerph19127482

**Published:** 2022-06-18

**Authors:** Nunzia Linzalone, Gabriele Donzelli, Maria Aurora Morales, Federico Vozzi

**Affiliations:** 1 Institute of Clinical Physiology of the National Research Council (CNR-IFC), 56124 Pisa, Italy; linunzia@ifc.cnr.it (N.L.); morales@ifc.cnr.it (M.A.M.); vozzi@ifc.cnr.it (F.V.); 2 Department of Health Sciences, University of Florence, 50134 Florence, Italy

**Keywords:** electrocardiogram examination, myocardial contractility, cardiovascular diseases, chronic exposure, ALTERNATIVE project, protocol

## Abstract

The association between environmental exposure to toxic substances and cardiovascular diseases (CVDs) in humans is widely recognized. However, the analysis of underlying pathophysiological mechanisms is essential to target meaningful endpoints of cardiotoxicity and allow a close-to-real life understanding of the role of chronic and acute exposure to multiple toxicants. The aim of this study is to outline the process for a systematic review of the literature that investigates the relationship between environmental pollution and left ventricular dysfunction. This systematic review and meta-analysis protocol will follow the Preferred Reporting Items for Systematic Reviews and Meta-Analyses (PRISMA-P) statement. PubMed, Embase, and Web of Science databases will be searched without applying search filters. Two independent reviewers will screen all titles and abstracts and identify the articles to be included in the synthesis. The risk of bias (RoB) will be assessed using an instrument developed for non-randomized (i.e., observational) studies (NRS) of environmental exposures. The results of cohort, case-control, cross-sectional, time-series, and case-crossover studies will be extracted and presented in tables considering different population subgroups and length of exposure. This protocol will be expected to provide a sound basis for selecting toxic chemicals and pollutants to contribute with the epidemiological evidence to the in vitro testing protocol within the EU-funded ALTERNATIVE Project.

## 1. Introduction

Different sources of potentially harmful substances of anthropogenic origin, including widespread and persistent chemical compounds, e.g., heavy metals, organochlorine pesticides, organic solvents, air pollutants, as well as pharmaceutical drugs and their metabolites, are recognized. Their presence in the environment is a source of great concern as potential causes of multi-organ damage due to their possible additive effects occurring inside the human body. Evidence-based literature, provided by a large number of studies, supports the negative role of environmental contaminants on human health [1]. Air, water, soil, and noise pollution have been extensively explored, and the health effects on humans were reported and also summarized in reviews and meta-analyses [2]. The steady increase over time of pollutant concentrations in the environment has turned into a threat to the health of an increasingly larger number of exposed populations, unequally affecting vulnerable individuals. Among public health topics, the prevention of cardiovascular morbidity/mortality is of primary relevance accounting for most of the deaths due to non-communicable diseases (NCDs) [3] (p. 10).

Cardiovascular diseases (CVDs) are the leading cause of death globally. In 2019, an estimated 17.9 million people died from CVDs, representing 32% of premature deaths mainly due to heart attack and stroke, particularly in the middle- and low-income countries [4]. The risk of developing cardiovascular diseases is strongly associated with exposure to environmental stressors such as pollutants in the air and noise level [5]. In polluted and densely populated urban areas, recognizing early warning signs of myocardial damage in chronically exposed individuals is essential to develop counteractive measures of primary prevention [6]. Additionally, due to the demographic transition, the incidence of heart failure due to the impairment of left ventricular contractility will inevitably rise with the aging of the population [7]; therefore, identifying potential triggers of left ventricular dysfunction in the environment may have a high impact on the health system.

The associations of CVDs with avoidable exposures, other than those due to behaviors such as tobacco smoke or alcohol consumption, are well established for many categories of toxic by-products from human activities (e.g., traffic, industries, waste disposal, energy production from fossil fuels) [8] and manufactured products as Persistent Organic Pollutants (POPs) which accumulate in the environment [9]. However, the increasing incidence and prevalence of cardiovascular diseases (CVDs) has attracted researchers’ interest in providing new evidence on the direct effects of the physical–chemical environment on myocardial damage. Individuals are exposed to mixtures of pollutants simultaneously entering the body through different routes as inhalation, ingestion, and skin (or eye) contact, making it difficult to acquire an in-depth comprehension of the risks of CVDs due to multi-organ toxicity effects. In fact, once a toxic agent enters the body, one or more organs and tissues start a signaling cascade and exhibit physiological responses, initially progressing to subclinical damage and ultimately leading to overt clinical disease [10]. Before heart failure occurs, toxicant-induced alterations lead to ventricular changes, such as focal myocyte loss and fibrotic replacement, as well as compensatory hypertrophy, leading to focally thickened ventricular walls. These myocardial structural changes lead to a decrease in the ability of myocytes to contract with enough sufficient speed and force to maintain the cardiac output necessary to meet body needs. The reduced left ventricular function may progress towards systolic heart failure (HF) [11].

Nowadays, abnormalities of left ventricular contractility due to a direct effect of toxic agents on the heart are not fully understood. Therefore, we aim to systematically review the literature dealing with left ventricular dysfunction in humans due to the environmental exposure to toxic substances. This systematic review will represent an essential part of the knowledge foundation of the ALTERNATIVE project (environmentAL Toxicity chEmical mixtuRes through aN innovative platform based on aged cardiac tissue models) granted under the European HORIZON 2020 program [12]. The project aims to develop an innovative platform collecting new knowledge on the cardiotoxicity of chemicals and their bio-transformation products. An innovative three-dimensional tissue engineering in vitro model, mimicking the human cardiac tissue, will provide the test for a reliable high-throughput monitoring system of physiological response to selected toxic agents based on multi-omics analyses and integrated into a Machine Learning (ML) risk assessment tool.

## 2. Materials and Methods

### 2.1. Objective

The present protocol will allow us to systematically review the effects of exposure to environmental pollutants on left ventricular dysfunction, given the following PECOS question (Population, Exposure, Comparator, and Study design) [13].

What is the existing evidence provided by observational and ecological studies (S) on the effect of exposure to environmental pollutants (E) on left ventricular dysfunction (O) compared to non-exposure, or low-exposure (C) in humans, including the susceptible groups of children and the elderly (P)?

### 2.2. Information Source and Search Strategy

PubMed [14], Embase [15], and Web of Science [16] will be searched without applying search filters, such as date or language restrictions. The following syntax will be used: (“heart failure” OR “left ventricular failure” OR “left ventricular function” OR “myocardial damage” OR “heart rate” OR “cardiotoxicity” OR “cardiac function” OR “cardiac dysfunction” OR “cardiovascular function” OR “cardiovascular dysfunction”) AND (“environmental exposur*” OR “joint toxic action” OR “Persistent Organic Pollutants” OR “POPs” OR “chemical* mixture*” OR “xenobiotic*” OR “persistent contaminants” OR “cadmium” OR “methylmercury” OR “pollut*” OR “particulate matter” OR “metal*” OR “mercury” OR “arsenic” OR “chromium” OR “CrVI” OR “pharmaceutical*” OR “pesticide*” OR “organic solvents” OR “exposure to lead” OR “lead exposure”) AND (“observational study” OR “epidemiological evidence” OR “cross-sectional” OR “case-control” OR “case-crossover” OR “time-series” OR “cohort” OR “follow-up studies” OR “odds ratio” OR “prospective” OR “epidemiol*” OR “residential”). Detailed search strategies used in PubMed, EMBASE and Web of Science are shown in Table S1.

Moreover, a check of the reference lists of relevant studies will be carried out to detect other studies to be included in the systematic review and inform the interpretation of findings [17].

### 2.3. Inclusion and Exclusion Criteria

Cohort, case-control, cross-sectional, time-series, and case-crossover original studies on the association between environmental exposure and cardiovascular diseases will be included. Mortality and hospitalization due to heart failure, hypertensive heart disease, and acute myocarditis will be considered, as well as admission to an outpatient clinic and diagnosis of left ventricular dysfunction. More specifically, we will consider the following specified conditions: combined systolic (congestive) and diastolic (congestive) heart failure (I50.4); systolic (congestive) heart failure (I50.2); hypertensive heart disease with heart failure (I11.0); dilated cardiomyopathy (I42.0); and cardiomyopathy unspecified (I42.9). Respect to exposures, pharmaceuticals, air pollution, metals, POPs including pesticides, industrial chemicals, and organic solvents will be considered. Studies investigating the effects on the general adult population and vulnerable groups (elderly, workers categories) will be included.

Randomized clinical trials, reviews, systematic reviews, editorials, commentaries, or other nonoriginal reports will be excluded. Also, in vivo and in vitro studies on cells, tissues, and animals will not be considered. Studies investigating the effects on susceptible groups (pre-existent cardiovascular diseases and/or the presence of documented lifestyle risk factors) and hospital readmission will be excluded.

### 2.4. Screening Process and Study Selection

Titles and abstracts will be imported into the Rayyan review management program [18]. After removing duplicate records, two investigators (NL and GD) will independently screen all titles and abstracts to decide which records meet the inclusion criteria and proceed to the full-text screening process. It will also be possible to add the label Maybe to any articles for which there is indecision. The screening process will be blinded, and a third investigator, an expert cardiologist (MAM), will solve any disagreement. After the initial screening process, articles will be downloaded for full-text screening. Similar to the previous phase, the two investigators will independently screen the full-text articles for inclusion, and the third investigator will solve any disagreement. The investigators will report the specific reason that articles are added to the PRISMA flow diagram [19].

### 2.5. Data Collection

After the full-text articles screening process and identifying the final studies for inclusion in the systematic review, a data collection form will be built. The two investigators will report all the relevant information for each study, and the third investigator will resolve any disagreement. More specifically, for each included study, the following information will be extracted: last name of the first author and year of publication, geographic location of the study area, study design, population type, sample size, environmental risk factor, exposure unit or exposure comparator, exposure temporality, health outcomes, risk estimations and their confidence intervals and p values, and the name of the journal. Regarding risk estimations, we will collect all the estimates reported by different models, e.g., those unadjusted and those adjusted.

### 2.6. Study and Evidence Quality Assessment

The two investigators will independently assess the RoB at the study-level using an instrument developed for NRSs (i.e., observational) addressing environmental exposures [20]. More specifically, the RoB will be assessed in the domains: (1) bias due to confounding, (2) bias in selection of participants into the study, (3) bias in classification of interventions, (4) bias due to departures from intended interventions, (5) bias due to missing data, (6) bias in measurement of outcomes, and (7) bias in selection of reported results. Table 1 shows the risk of the bias matrix which will be used to assess the RoB of each study. Judgments for each RoB item can be: ‘Low RoB’, ‘Moderate RoB’, ‘Serious RoB’, or ‘Critical RoB’. Similarly, an overall judgment about the bias at the study level is either ‘Low RoB’, ‘Moderate RoB’, ‘Serious RoB’, or ‘Critical RoB’ [21].

### 2.7. Qualitative and Quantitative Synthesis of Results

**Figure 1:**
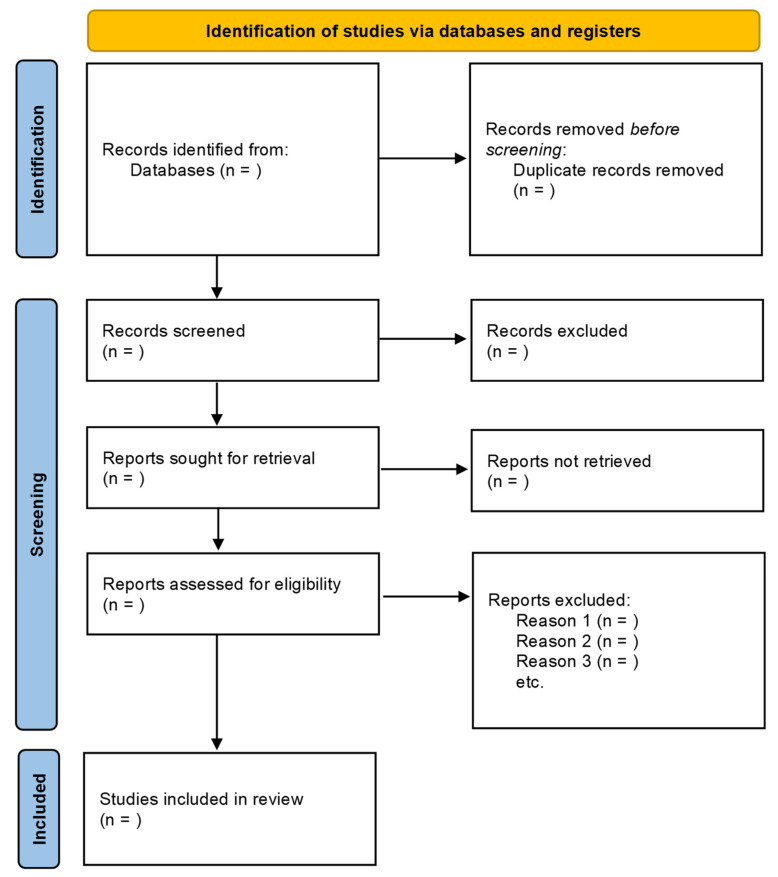
PRISMA 2020 generic flow diagram for new systematic reviews.

A PRISMA flow diagram (Figure 1) depicts the process of selection and figures of selected studies. It includes the items of the studies initially identified; the items excluded prior to the screening; the items excluded after the screening based on titles or titles and abstracts; the reports retrieved for detailed evaluation; potentially eligible reports that were not retrievable; the retrieved reports that did not meet inclusion criteria; and the primary reasons for exclusion. The key characteristics of each study, extracted in the phases of data collection, will be presented in summary tables to facilitate the comparison of characteristics across the studies.

The statistical method and the risk estimations will be reported for each study, and when applicable, the results will be stratified for different subgroups of the populations. The possibility of carrying out a meta-analysis will be evaluated based on the heterogeneity of the study designs in terms of types of chemical exposure, outcomes, and populations investigated. Similarly, across different studies, various types of effect measures are used, mainly odds ratios, risk ratios, and hazard ratios. In this case, before performing the meta-analysis, it will be necessary to conduct a homogenization procedure by converting the various effect measures into a single type of effect estimate. Subsequently, a random-effects model using the REsidual Maximum Likelihood (REML) method will be used to calculate the overall estimate [22]. Forest plots will be built to visualize the individual study estimates and confidence intervals.

Moreover, a subgroup analysis will be carried out by pollutant, study design, and location. Using Egger’s test, funnel plot asymmetry will assess the potential publication bias [23]. All statistical analysis will be carried out using RStudio Open Source Edition, Version 1.2.5001 (Boston, MA, USA) [24].

### 2.8. Protocol Registration

Following the Preferred Reporting Items for Systematic reviews and Meta-Analysis protocol (PRISMA-P) guidelines [25], our systematic review protocol was registered at the International Prospective Register of Systematic Reviews (PROSPERO) on 11 January 2022 with identifier CRD42022293484 [26].

## 3. Discussion

Systematic reviews and meta-analyses of medical literature have developed rapidly in the past decades, playing a key role in decision making in evidence-based medical practice [27]. Given the large number of studies on environmental health, review articles can also play an important role in improving the use of evidence in environmental policy-making [28]. The synthesis of research findings of primary studies, which address a specific environmental issue, may be very useful in obtaining stronger evidence and improving the knowledge in a specific research field. However, observational studies provide weaker evidence when compared to toxicity evaluation in clinical trials due to the inherent limitation of the epidemiologic approach [29]. Therefore, RoB tools are adequate to mitigate confounding and selection bias to overcome this limitation.

This systematic review protocol was developed specifically to increase the knowledge on harmful environmental pollutants that lead to the cardiovascular diseases as a basis to drive further research. In fact, there is mounting evidence of the positive association between some environmental pollutants and the onset of cardiovascular diseases. The evidence is well documented and some pollutants show a high health hazard, for example particulate matter less than 2.5 µg/m^3^ (PM 2.5). However, little is known about the mechanisms activated by exposure to environmental pollutants that induce damage to the myocardium and the reduction of its normal functionality. If this process is not recognized, it could lead to heart disease. We aim to assess the complex relationship that is modulated by different activation paths, and is exacerbated by high concentrations and cumulative exposures of pollutants with additive effects. Other risk factors and subjective characteristics contribute to the predisposition of or reduction of risk on an individual basis (e.g., age, gender, genetics). All those relevant parameters in the exposure–outcome relationship need to be considered appropriately by a stratified analysis for subgroups of variables. However, if the evidence is scarce, the systematic review will identify knowledge gaps [10] and the use of discrete categories of judgment will provide a consensus on the quality of the evidence. If, as desirable, further research will address the association between the left ventricular function and the impact of environmental pollutants, a new systematic review could be performed to assess the advancement of this knowledge. This protocol will therefore represent a tool to update and promote future research.

In the last 20 years, observational studies have improved our knowledge and awareness of the critical role of environmental pollution in causing damage to exposed populations, in affecting the incidence of cardiovascular diseases, and in mortality [6]. The cardiotoxicity of pollutants has been assessed and reviewed in terms of the associations of selected contaminants (measured at the individual level) with the risk of cardiovascular outcomes, including cardiovascular disease, coronary heart disease, and stroke. More specifically, ischemic heart disease and cardiac arrhythmias have been extensively documented about chronic exposure to different pollutants. However, it is still unclear how observational studies could help to define the missing link regarding toxicity results from in vitro/in vivo scientific knowledge. A systematic review will provide an initial answer to this question, which is currently under discussion among academics. Individual heterogeneity in progression towards a disease makes it difficult to understand the causal link between toxic compounds and the outcome. The proposed PECOS research question will introduce an observational point on the early phase of the disease, focusing on the relationship between myocardial damage and pollutants.

Within the ALTERNATIVE project, the integration of different lines of evidence from toxicology, in vitro tests and -omics will provide wide data to support the identification of the potential candidate Adverse Outcome Pathways (AOPs); in this context, the observational epidemiological studies are instrumental to the definition of the appropriate outcome among the cardiac diseases that can plausibly be traced back to a pollutant showing similar AOPs in the organism. A great part of published reviews provides health evidence on defined diseases and causes of death to advise public health interventions and regulations while little acknowledgement is attributed to preclinical and symptomatic health indicators, which could affect the largest portion of the population [30]. Data provided by this review could allow the understanding of initial modification in left ventricular function due to toxic exposure, thus focusing on early, possibly preclinical, endpoints.

## 4. Conclusions

A dedicated article will be drafted to reflect the significance and value of the research stemming from this research protocol application. The findings of the review will extend beyond previous systematic reviews, complementing the evidence on the relationship between environmental exposure and cardiovascular disease. We will review the available epidemiological studies to enlighten the association between contaminants and heart disease starting from the early signs of myocardial damage. The findings from this systematic review will attempt to provide a new insight on the role of environmental pollution and contractile dysfunction.

## Figures and Tables

**Table 1:** Risk of Bias matrix.

Studies	Confounding	Selection	Measurement of Exposure	Departures from Exposure	Missing Data	Measurement of Outcomes	Reported Results
1							
2							
…							
				Low	Moderate	Serious	Critical

## Supplementary Materials

The following supporting information can be downloaded at: https://www.mdpi.com/article/10.3390/ijerph19127482/s1, Table S1: Search strategy.

## References

[1] U.S. Environmental Protection Agency Exposure to Environmental Contaminants. https://www.epa.gov/report-environment/exposure-environmental-contaminants.

[2] Dominski F.H., Lorenzetti Branco J.H., Buonanno G., Stabile L., Gameiro da Silva M., Andrade A. (2021). Effects of air pollution on health: A mapping review of systematic reviews and meta-analyses. Environ. Res..

[3] Chapter 10_SOER2020. Chemical Pollution—European Environment Agency. https://www.eea.europa.eu/publications/soer-2020/chapter-10_soer2020-chemical-pollution/view.

[4] Cardiovascular Diseases (CVDs). https://www.who.int/news-room/fact-sheets/detail/cardiovascular-diseases-.

[5] Münzel T., Hahad O., Sørensen M., Lelieveld J., Duerr G.D., Nieuwenhuijsen M., Daiber A. (2021). Environmental risk factors and cardiovascular diseases: A comprehensive review. Cardiovasc. Res..

[6] Boyd R., McMullen H., Beqaj H., Kalfa D. (2022). Environmental Exposures and Congenital Heart Disease. Pediatrics.

[7] Strait J.B., Lakatta E.G. (2012). Aging-associated cardiovascular changes and their relationship to heart failure. Heart Fail. Clin..

[8] Kupcikova Z., Fecht D., Ramakrishnan R., Clark C., Cai Y.S. (2021). Road traffic noise and cardiovascular disease risk factors in UK Biobank. Eur. Heart J..

[9] Miguel Pérez-Carrascosa F., Gómez-Peña C., Echeverría R., Juan Jiménez Moleón J., Manuel Melchor J., García-Ruiz A., Luis Navarro-Espigares J., Cabeza-Barrera J., Martin-Olmedo P., Carlos Ortigosa-García J. (2021). Historical exposure to persistent organic pollutants and cardiovascular disease: A 15-year longitudinal analysis focused on pharmaceutical consumption in primary care. Environ. Int..

[10] Cosselman K.E., Navas-Acien A., Kaufman J.D. (2015). Environmental factors in cardiovascular disease. Nat. Rev. Cardiol..

[11] Schwinger R.H.G. (2021). Pathophysiology of heart failure. Cardiovasc. Diagn..

[12] environmentAL Toxicity chEmical mixtuRes through aN Innovative Platform Based on Aged Cardiac Tissue Model|ALTERNATIVE Project|Fact Sheet|H202 |CORDIS|European Commission. https://cordis.europa.eu/project/id/101037090.

[13] Morgan R.L., Whaley P., Thayer K.A., Schünemann H.J. (2018). Identifying the PECO: A framework for formulating good questions to explore the association of environmental and other exposures with health outcomes. Environ. Int..

[14] PubMed https://pubmed.ncbi.nlm.nih.gov/.

[15] Embase https://www.embase.com/landing?status=grey.

[16] Document Search—Web of Science Core Collection. https://www.webofscience.com/wos/woscc/basic-search.

[17] Horsley T., Dingwall O., Sampson M. (2011). Checking reference lists to find additional studies for systematic reviews. Cochrane Database Syst. Rev..

[18] Ouzzani M., Hammady H., Fedorowicz Z., Elmagarmid A. (2016). Rayyan—a web and mobile app for systematic reviews. Syst. Rev..

[19] PRISMA http://prisma-statement.org/prismastatement/flowdiagram.aspx.

[20] Morgan R.L., Thayer K.A., Santesso N., Holloway A.C., Blain R., Eftim S.E., Goldstone A.E., Ross P., Guyatt G., Schünemann H.J. (2018). Evaluation of the risk of bias in non-randomized studies of interventions (ROBINS-I) and the ‘target experiment’ concept in studies of exposures: Rationale and preliminary instrument development. Environ. Int..

[21] Morgan R.L., Thayer K.A., Santesso N., Holloway A.C., Blain R., Eftim S.E., Goldstone A.E., Ross P., Ansari M., Akl E.A. (2019). A risk of bias instrument for non-randomized studies of exposures: A users’ guide to its application in the context of GRADE. Environ. Int..

[22] Normand S.-L.T. (1999). Meta-analysis: Formulating, evaluating, combining, and reporting. Stat. Med..

[23] Egger M., Smith G.D., Schneider M., Minder C. (1997). Bias in meta-analysis detected by a simple, graphical test. BMJ.

[24] RStudio Team (2019). RStudio: Integrated Development for R. RStudio, Inc., Boston, MA.

[25] Shamseer L., Moher D., Clarke M., Ghersi D., Liberati A., Petticrew M., Shekelle P., Stewart L.A. (2015). Preferred reporting items for systematic review and meta-analysis protocols (PRISMA-P) 2015: Elaboration and explanation. BMJ.

[26] Chien P.F.W., Khan K.S., Siassakos D. (2012). Registration of systematic reviews: PROSPERO. BJOG.

[27] Gopalakrishnan S., Ganeshkumar P. (2013). Systematic Reviews and Meta-analysis: Understanding the Best Evidence in Primary Healthcare. J. Fam. Med. Prim. Care.

[28] Collins A.M., Coughlin D., Randall N. (2019). Engaging environmental policy-makers with systematic reviews: Challenges, solutions and lessons learned. Environ. Evid..

[29] Vedal S., Campen M.J., McDonald J.D., Larson T.V., Sampson P.D., Sheppard L., Simpson C.D., Szpiro A.A. (2013). National Particle Component Toxicity (NPACT) initiative report on cardiovascular effects. Res. Rep. Health Eff. Inst..

[30] Rojas-Rueda D., Morales-Zamora E., Alsufyani W.A., Herbst C.H., AlBalawi S.M., Alsukait R., Alomran M. (2021). Environmental Risk Factors and Health: An Umbrella Review of Meta-Analyses. Int. J. Environ. Res. Public Health.

