# Supplementary material for: Protocol for a Systematic Review and Meta-Analysis of Observational Studies on the Association of Exposure to Toxic Environmental Pollutants and Left Ventricular Dysfunction
Source: Int J Environ Res Public Health. 2022 Jun 18;19(12):7482. doi: 10.3390/ijerph19127482 (PMC9223820; doi:10.3390/ijerph19127482)
Supplement: Supplementary file 1 [file ijerph-19-07482-s001.zip › ijerph-1724196-supplementary.pdf]

Table S1: Search strategy

Database	Search string
**PubMed**	("heart failure"[Title/Abstract] OR "left ventricular failure"[Title/Abstract] OR "left ventricular function"[Title/Abstract] OR "myocardial damage"[Title/Abstract] OR "heart rate"[Title/Abstract] OR "cardiotoxicity"[Title/Abstract] OR "cardiac function" [Title/Abstract] OR "cardiac dysfunction" [Title/Abstract] OR "cardiovascular function" [Title/Abstract] OR "cardiovascular dysfunction" [Title/Abstract]) AND (“environmental exposur*"[Title/Abstract] OR “joint toxic action” [Title/Abstract] OR “Persistent Organic Pollutants” [Title/Abstract] OR “POPs” [Title/Abstract] OR “chemical* mixture*” [Title/Abstract] OR “xenobiotic*” [Title/Abstract] OR "persistent contaminants"[Title/Abstract] OR "cadmium"[Title/Abstract] OR "methylmercury"[Title/Abstract] OR “pollut*"[Title/Abstract] OR "particulate matter"[Title/Abstract] OR "metal*"[Title/Abstract] OR "mercury"[Title/Abstract] OR "arsenic"[Title/Abstract] OR "chromium"[Title/Abstract] OR "CrVI"[Title/Abstract] OR "pharmaceutical*"[Title/Abstract] OR "pesticide*"[Title/Abstract] OR "organic solvents"[Title/Abstract] OR "exposure to lead" OR "lead exposure"[Title/Abstract]) AND ("observational study"[Title/Abstract] OR "epidemiological evidence"[Title/Abstract] OR "cross-sectional"[Title/Abstract] OR "case-control"[Title/Abstract] OR "case-crossover"[Title/Abstract] OR "time-series"[Title/Abstract] OR "cohort"[Title/Abstract] OR "follow-up studies"[Title/Abstract] OR "odds ratio"[Title/Abstract] OR "prospective"[Title/Abstract] OR "epidemiol*"[Title/Abstract] OR "residential"[Title/Abstract])
**Embase**	('heart failure':ti,ab,kw OR 'left ventricular failure':ti,ab,kw OR 'left ventricular function':ti,ab,kw OR 'myocardial damage':ti,ab,kw OR 'heart rate':ti,ab,kw OR 'cardiotoxicity':ti,ab,kw OR 'cardiac function':ti,ab,kw OR 'cardiac dysfunction':ti,ab,kw OR 'cardiovascular dysfunction':ti,ab,kw OR 'cardiovascular function':ti,ab,kw) AND ('environment exposur*':ti,ab,kw OR 'joint toxic action':ti,ab,kw OR 'Persistent Organic Pollutants':ti,ab,kw OR 'POPs':ti,ab,kw OR 'chemical* mixture*':ti,ab,kw OR 'xenobiotic*':ti,ab,kw OR 'persistent contaminants':ti,ab,kw OR 'cadmium':ti,ab,kw OR 'methylmercury':ti,ab,kw OR 'pollut*':ti,ab,kw OR 'particulate matter':ti,ab,kw OR 'metal*':ti,ab,kw OR 'mercury':ti,ab,kw OR 'arsenic':ti,ab,kw OR 'chromium':ti,ab,kw OR 'CrVI':ti,ab,kw OR 'pharmaceutical*':ti,ab,kw OR 'pesticide*':ti,ab,kw OR 'organic solvents':ti,ab,kw OR 'exposure to lead':ti,ab,kw OR 'lead exposure':ti,ab,kw) AND ('observational study':ti,ab,kw OR 'epidemiological evidence':ti,ab,kw OR 'cross-sectional':ti,ab,kw OR 'case-control':ti,ab,kw OR 'case-crossover':ti,ab,kw OR 'time-series':ti,ab,kw OR 'cohort':ti,ab,kw OR 'follow-up studies':ti,ab,kw OR 'odds ratio':ti,ab,kw OR 'prospective':ti,ab,kw OR 'epidemiol*':ti,ab,kw OR 'residential':ti,ab,kw)
**Web of Science**	("heart failure" OR "left ventricular failure" OR "left ventricular function" OR "myocardial damage" OR "heart rate" OR "cardiotoxicity" OR "cardiac function" OR "cardiac dysfunction" OR "cardiovascular function" OR "cardiovascular dysfunction") AND (“environmental exposur*" OR “joint toxic action” OR “Persistent Organic Pollutants” OR “POPs” OR “chemical* mixture*” OR “xenobiotic*” OR "persistent contaminants" OR "cadmium" OR "methylmercury" OR “pollut*" OR "particulate matter" OR "metal*" OR "mercury" OR "arsenic" OR "chromium" OR "CrVI" OR "pharmaceutical*" OR "pesticide*" OR "organic solvents" OR "exposure to lead" OR "lead exposure") AND ("observational study" OR "epidemiological evidence" OR "cross-sectional" OR "case-control" OR "case-crossover" OR "time-series" OR "cohort" OR "follow-up studies" OR "odds ratio" OR "prospective" OR "epidemiol*" OR "residential")
